# Mode division multiplexing reconstructive spectrometer with an all-fiber photonics lantern

**DOI:** 10.1007/s12200-024-00130-6

**Published:** 2024-07-17

**Authors:** Junrui Liang, Jun Ye, Xiaoya Ma, Yao Lu, Jun Li, Jiangming Xu, Zilun Chen, Jinyong Leng, Zongfu Jiang, Pu Zhou

**Affiliations:** 1 College of Advanced Disciplinary Studies, National University of Defense Technology, Changsha, 410073 China; 2 Nanhu Laser Laboratory, National University of Defense Technology, Changsha, 410073 China; 3 Hunan Provincial Key Laboratory of High Energy Laser Technology, National University of Defense Technology, Changsha, 410073 China; 4 Space Engineering University, Beijing, 101416 China

**Keywords:** High-accuracy, Resolution enhancement, Reconstructive spectrometer, Mode division multiplexing, Photonics lantern

## Abstract

This study presents a high-accuracy, all-fiber mode division multiplexing (MDM) reconstructive spectrometer (RS). The MDM was achieved by utilizing a custom-designed 3 × 1 mode-selective photonics lantern to launch distinct spatial modes into the multimode fiber (MMF). This facilitated the information transmission by increasing light scattering processes, thereby encoding the optical spectra more comprehensively into speckle patterns. Spectral resolution of 2 pm and the recovery of 2000 spectral channels were accomplished. Compared to methods employing single-mode excitation and two-mode excitation, the three-mode excitation method reduced the recovered error by 88% and 50% respectively. A resolution enhancement approach based on alternating mode modulation was proposed, reaching the MMF limit for the 3 dB bandwidth of the spectral correlation function. The proof-of-concept study can be further extended to encompass diverse programmable mode excitations. It is not only succinct and highly efficient but also well-suited for a variety of high-accuracy, high-resolution spectral measurement scenarios.

**Graphical Abstract:**

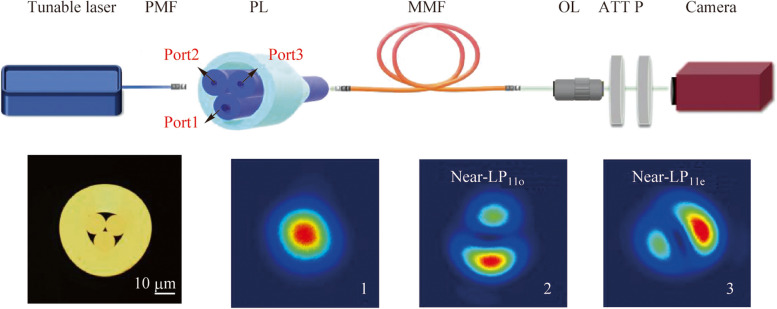

## Introduction

Spectrometers play a crucial role in various industries and scientific fields, such as biology, astronomy, medical treatment, and laser beam characterization [1–3]. For traditional bench-top spectrometers, a contradiction always exists between the resolution and the size. In the past decade, there has been growing interest in speckle reconstructive spectrometers (RSs) because of their small footprint and exceptional spectral resolution [4–6]. Speckle RSs work by mapping spectral signals to spatial intensity patterns through scattering mediums [7–9], among which utilizing the interference among guided modes in a multimode fiber (MMF) is an attractive option [10–13].

Speckle RSs using MMFs offer benefits like enhanced resolution and compact design, but they also encounter the pressing need to enhance accuracy. Accuracy serves as a critical performance metric for spectrometers. In traditional spectrometers, accuracy is contingent on well-established factors such as the quality of dispersion components, detector performance, and optical path alignment [14]. However, improving accuracy in speckle RSs remains a significant challenge. Among the various existing approaches, the spectra are always encoded into a speckle pattern using the transmission matrix (TM) of the MMF, followed by a decoding algorithm that reconstructs the spectra [15]. The relationship between observed speckle intensity **I**, transmission matrix **T**, and spectrum **S** can be expressed as **I = T·S**. Nevertheless, spectral encoding often experiences information loss, hindering advancements in reconstruction accuracy. To mitigate the information loss, there has been a consistent focus on expanding the number of independent spatial channels of the observed intensity **I** [16, 17]. To achieve this, multiplexing techniques are promising solutions, which have been extensively applied in various fields [18, 19]. Wavelength division multiplexing [20] and space division multiplexing [21] techniques have been used in RSs. In Ref. [21], the input spatial degrees of freedom were exploited by using a multicore fiber. Actually, spatial degrees of freedom can also be exploited by using modes. The idea of switching the repeatable input launch condition to enhance independent spatial channels has been employed in a silicon multimode waveguide RS, leading to an expanded faithful operating bandwidth [22]. Simulation studies have been conducted on miniaturized RS based on mode division multiplexing (MDM) [23], but the resolution achieved was limited to several nanometers. The implementation of an all-fiber MDM system is anticipated to improve both the resolution and the spectral reconstruction accuracy of RSs.

MDM-based RSs have been also reported to possess spectral resolution enhancement capability [23]. In the study, initially, there were not enough observed spatial channels (dozens) to match the spectral channels (hundreds) that needed to be reconstructed. This lack of data on photocurrent detection made the ‘electronic signatures’ for each wavelength unclear. By increasing the amount of photocurrent detection using MDM, the ‘electronic signatures’ for each wavelength became clearer, thereby improving the resolution. However, when the amount of observation data is no longer a limiting factor, such as the observation data of speckle RSs coming from high-pixel cameras, the enhancement of resolution indeed poses a challenging problem. Speckle RSs operate on the principle that the phase delay between guided modes is wavelength-dependent. Resolution improvement can be routinely achieved by exciting higher-order modes and increasing the length of the waveguide. A new resolution enhancement mechanism independent of these two methods was introducing polarization modulation for speckles, thus reducing correlation between speckles at adjacent wavelengths [3]. However, this passive modulation is fixed once the magneto-optical material is selected. MDM technology may offer an alternative resolution enhancement scheme based on active mode modulation.

One of the key devices for the MDM system is the mode multiplexer. The all-fiber photonics lantern, with its advantages of low loss and minimal crosstalk, presents an attractive solution. The all-fiber photonics lantern has been employed in communication capacity expansion [24], computational optical imaging [25], spatial mode control [26], and so on [27]. Considering its excellent compatibility with existing all-fiber RSs, we chose the all-fiber photonics lantern as the mode multiplexer.

In this paper, we presented a high-accuracy speckle RS, in which a homemade all-fiber photonics lantern was employed to achieve MDM. By exciting the MMF with various spatial modes, distinct scattering processes were generated. It facilitated the expansion of transmissible information capacity, allowing for more comprehensive encoding from spectral information to speckle patterns. Spectral resolution of 2 pm was achieved and 2000 spectral channels were able to be recovered. In contrast to schemes using single mode and two modes for excitation, the three-mode-excitation approach exhibited higher spectral reconstruction accuracy. Additionally, we proposed a resolution enhancement method based on active mode modulation, exploiting the abrupt differences in output speckle caused by the incident modes. Our scheme was characterized by its simplicity, effectiveness, and suitability for a wide range of high-accuracy, high-resolution RS applications.

## Experimental setup

**Fig. 1:**
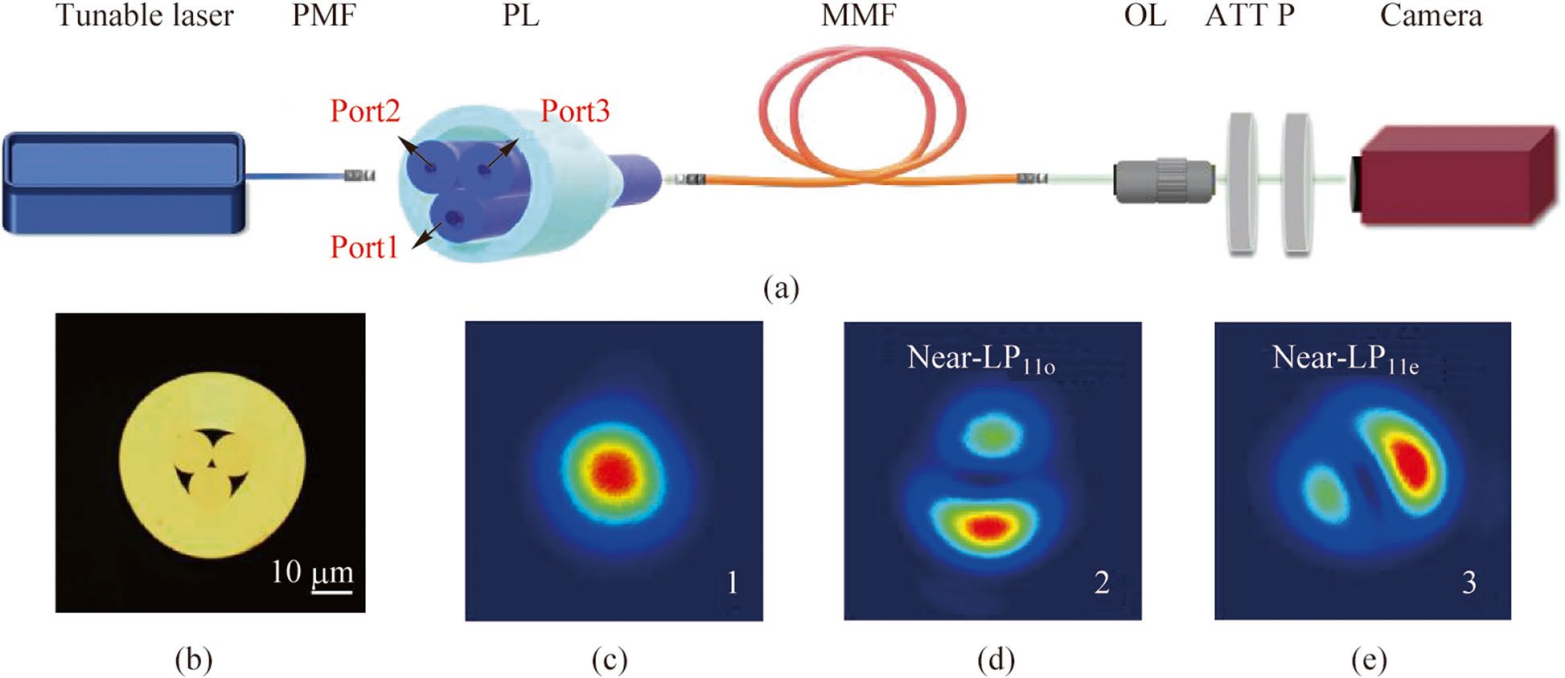
**a** Experimental setup of the all-fiber RS based on MDM technology. PMF: polarization-maintaining fiber; PL: photonics lantern; MMF: multimode fiber; OL: objective lens; ATT: attenuator; P: polarizer. **b** Optical microscope of the fiber-tube bundle end face of the homemade 3 × 1 mode-selective photonics lantern (scale bar = 10 μm). Output the mode distributions at the output fiber end with a 30 µm core diameter, corresponding to input **c** arm 1, **d** arm 2, and **e** arm 3

The experimental setup of the proposed all-fiber MDM RS is shown in Fig. 1a. A tunable laser (linearly polarized, tuning range: 1520–1567 nm, 3 dB linewidth: 5 MHz) was used to allow precise wavelength shifts for calibration, in conjunction with a piece of polarization-maintaining fiber (PMF). The PMF segment used here ensured that the light waves injected to subsequent RS was linearly polarized during calibration. After calibration, the light to be detected was coupled in the RS with an in-line fiber polarizer and a PMF. The PMF was then fused with a homemade 3 × 1 mode-selective photonics lantern, fabricated by tapering a bundle of fibers inside a low-index tube [26]. Figure 1b depicts the optical microscope of the fiber-tube bundle end face of the photonics lantern. The input single-mode fibers of the photonics lantern had varying core diameters, resulting in different output modes for each. Figure 1c–e present the single-arm testing results of the 3 × 1 mode-selective photonics lantern. When injecting the fundamental mode field from arm 1 with a core diameter of 15 µm, a larger mode area LP_01_ mode was obtained at the output end with a core diameter of 30 µm. When injecting the fundamental mode field from arm 2 with a core diameter of 10 µm, a near-LP_11o_-mode was obtained at the output end. When injecting the fundamental mode field from arm 3 with a core diameter of 10 µm, a near-LP_11e_-mode was correspondingly attained.

In another work on speckle RS [28], a planar-integrated photonics lantern was used to sample the modal field at the output end. In contrast, the photonics lantern used here is to modify the launching conditions at the input end. By switching the input arms of the photonics lantern, light waves with different mode characteristics were launched into a segment of step-index MMFs (length = 100 m, core diameter = 105 μm, numerical aperture = 0.22). The temporal stability of the same type and length of MMF can be referenced in another research on speckle RSs [13]. Although long MMFs are sensitive to environmental conditions, numerous solutions have been proposed to address this issue, including stringent temperature control and stabilization [11], wavelength correction [10], and deep learning [13]. Due to the long optical path, interference occurred between the guided modes in the multi-mode optical fiber, resulting in wavelength-dependent speckle patterns. These near-field speckle patterns were amplified using a 50 × objective lens and captured by an infrared camera with pixel size of 20 µm × 20 µm. Moreover, an attenuator was used to avoid saturation of the camera before image acquisition, and a polarizer was employed to enhance the contrast.

During the calibration phase, the tunable laser wavelength was systematically adjusted in increments of 1 pm over a range of 2 nm. This scanning procedure resulted in a spectral sequence consisting of 2000 channels, keeping the order of magnitude the same as the other schemes focused on broadband operation [20, 21, 29]. The tunable laser for calibration was able to switch wavelengths at a rate of 10 kHz, and the camera used for capturing speckles operates at approximately 200 Hz. Therefore, recalibrating the transmission matrix for this task would take about 10 s, making it a viable solution to mitigate the temporal instability of the proposed RS. Subsequently, during the measurement, the speckle pattern of the target spectra was synthesized by summing the calibrated speckle patterns across various wavelengths [16]. The spectra were then reconstructed by employing a combination of the truncated inversion technique and a nonlinear optimization algorithm mentioned in Ref. [16].

## Results and discussion

**Fig. 2:**
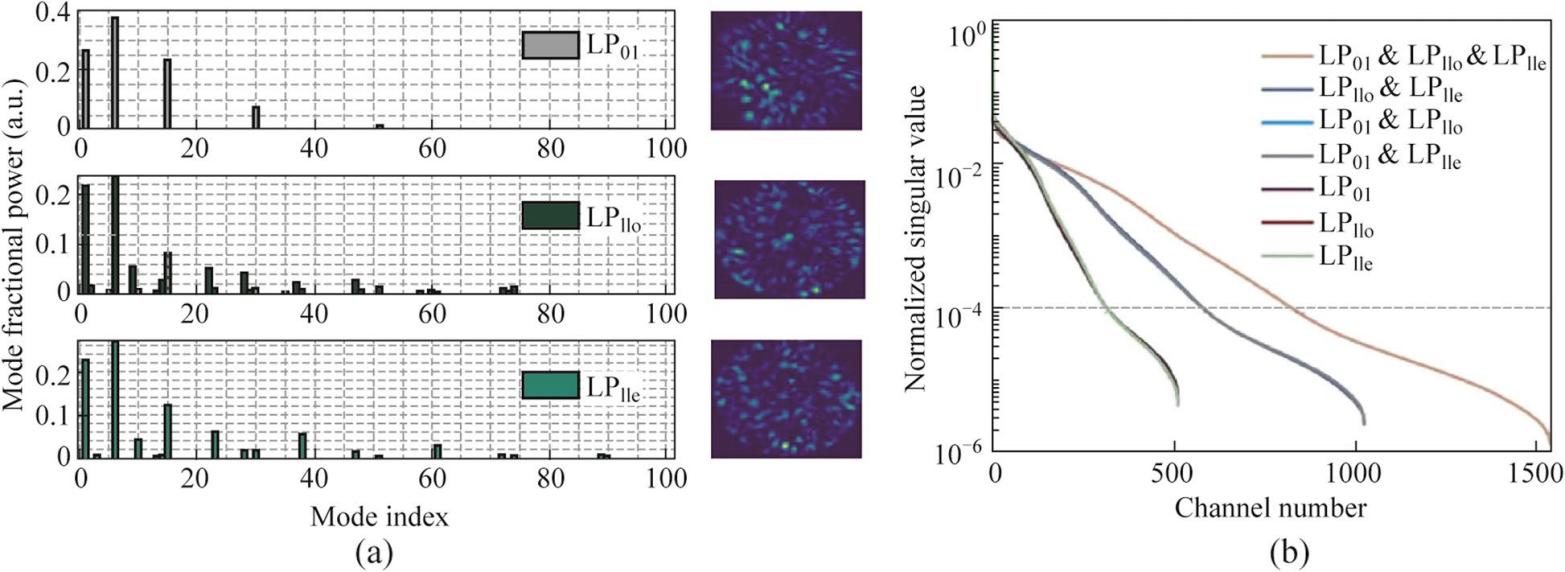
**a** Power distribution calculation results of the first 100 modes in the MMF under three mode excitation conditions, along with the corresponding MMF output speckle patterns. **b** NSVs corresponding to seven exciting combinations were measured. Before performing the singular value decomposition, the measured matrices underwent normalization. The singular values were then arranged in descending order according to their magnitudes

The speckle field at the MMF output end is a function of the spatial mode field at the input end. Given the electric field of the exciting beam at the incident plane of the MMF, **e**_inc_(*u,v*), and the electric field of the LP_mn_ mode in the MMF, **e**_mn_(*u,v*), the normalized power coupling coefficient can be expressed as:

1 $$\eta_{{{\text{mn}}}} = \frac{{\left| {\iint\limits_{{A_{{{\text{core}}}} }} {e_{{{\text{inc}}}} (u,v) \cdot e_{{{\text{mn}}}}^{*} (u,v){\text{d}}u{\text{d}}v}} \right|^{2} }}{{\iint\limits_{{A_{{{\text{core}}}} }} {\left| {e_{{{\text{inc}}}} (u,v)} \right|^{2} {\text{d}}u{\text{d}}v\iint\limits_{{A_{{{\text{core}}}} }} {\left| {e_{{{\text{mn}}}} (u,v)} \right|^{2} {\text{d}}u{\text{d}}v}}}},$$

where *A*_core_ represents the mode field area of the MMF. * indicates the conjugate operation. *η*_mn_ signifies that *η*_mn_ times the power of the incident beam is injected into the LP_mn_ mode. Considering the three output beams of the photonics lantern measured in Fig. 1 for excitation, we calculated the power distribution of the first 100 excited LP modes in the MMF for each of the three cases, as shown in the left column of Fig. 2a. The mode index here was sorted in descending order based on the effective refractive index of LP modes. Specifically, the first LP mode was the LP_01_ mode, and the 100th mode was LP_45o_. More detailed correspondences can be referred to Ref. [30]. Due to differences in the initially excited mode weights, the subsequent mode coupling and power diffusion in the MMF would be completely different. The speckle patterns at the MMF output for the three cases are shown in the right column of Fig. 2a.

By altering the incident modes, an expanded and improved encoder was obtained to establish the relationship from the original spectra to the observed intensity data. To assess the superiority of the MDM-based encoding scheme, we calculated the independent spatial channel number of its TM, which can be represented by the number of nonzero singular values (NSV), as detailed in Ref. [28]. Our MDM system was able to generate three types of speckles, corresponding to the LP_01_ mode excitation, LP_11o_ mode excitation, and LP_11e_ mode excitation. Among these three types of speckles, seven combinations could be chosen for spectral reconstruction.

Figure 2b presents the number of NSV of these seven combinations. When the same amount of speckle, e.g., one or two, was used for reconstruction, different types of launching modes did not significantly vary the NSV. Therefore, in the latter text, it is unnecessary to choose an optimal combination when conducting the comparison experiment of single-mode-excitation and two-mode-excitation. However, as the amount of speckle data used for reconstruction increased, new launching modes could generate more NSV, i.e., more independent spatial channels, rather than redundant data repetitions.

**Fig. 3:**
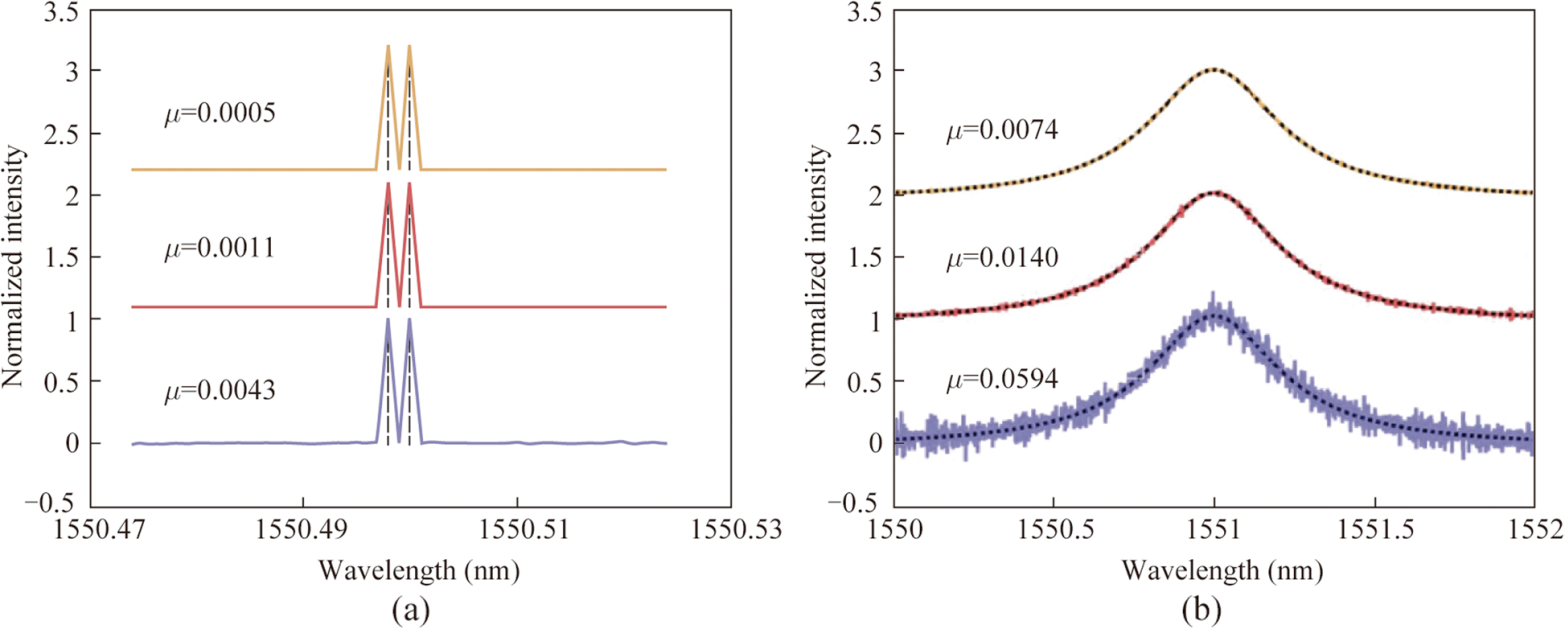
**a** Spectrum with two narrow peaks separated by 2 pm (blacked dashed line) and its reconstructed results by using single-mode-excitation (purple solid line), two-mode-excitation (red solid line), and three-mode-excitation (yellow solid line). **b** Lorentzian spectrum with FWHM linewidth of 0.5 nm (blacked dashed line) and its reconstructed results by using single-mode-excitation (purple solid line), two-mode-excitation (red solid line), and three-mode-excitation (yellow solid line)

The expanded speckles generated by MDM technology were used for spectral reconstruction. Without loss of generality, three cases corresponding to single-mode-excitation (LP_01_), two-mode-excitation (LP_01_ and LP_11o_), and three-mode-excitation (LP_01_, LP_11e_, and LP_11o_), were evaluated. In this paper, the measurement accuracy is characterized by the reconstructed error, *μ*, defined as the standard deviation between the ground truth and the recovered spectrum:

2 $$\mu = \sqrt {\frac{1}{M}\sum\nolimits_{i = 1}^{M} {(S_{i}^{{\text{g}}} - S_{i}^{{\text{r}}} )^{2} } },$$

where *M* represents the spectral channel number under test. The spectral resolution was evaluated by detecting two wavelengths separated by 2 pm. As shown in Fig. 3a, reconstruction errors of 0.0043, 0.0011, and 0.0005 were obtained for the respective cases. The signal-to-noise ratios (SNRs) were approximately 17, 23, and 27 dB, correspondingly. By using three distinct types of speckles corresponding to three different excitation modes, significantly lower recovery errors (88% and 55% reduction) and higher SNR values (10 and 4 dB improvement) were achieved, compared to methods relying on single or dual modes.

Moreover, to assess the accuracy of broadband signal reconstruction, a Lorentzian spectrum with a full width at half maximum (FWHM) linewidth of 0.5 nm was designated as the target. Figure 3b presents the recovered spectra obtained using the three approaches, along with the ground truth. While both the single-mode and two-mode excitation methods reconstructed spectra with varying levels of noise, the three-mode-excitation method demonstrated the closest agreement with the ground truth. The reconstruction error of the three-mode-excitation method was determined to be 0.0074, which is 88% and 47% lower than that of the two previous methods (0.0594 and 0.0140), respectively.

**Fig. 4:**
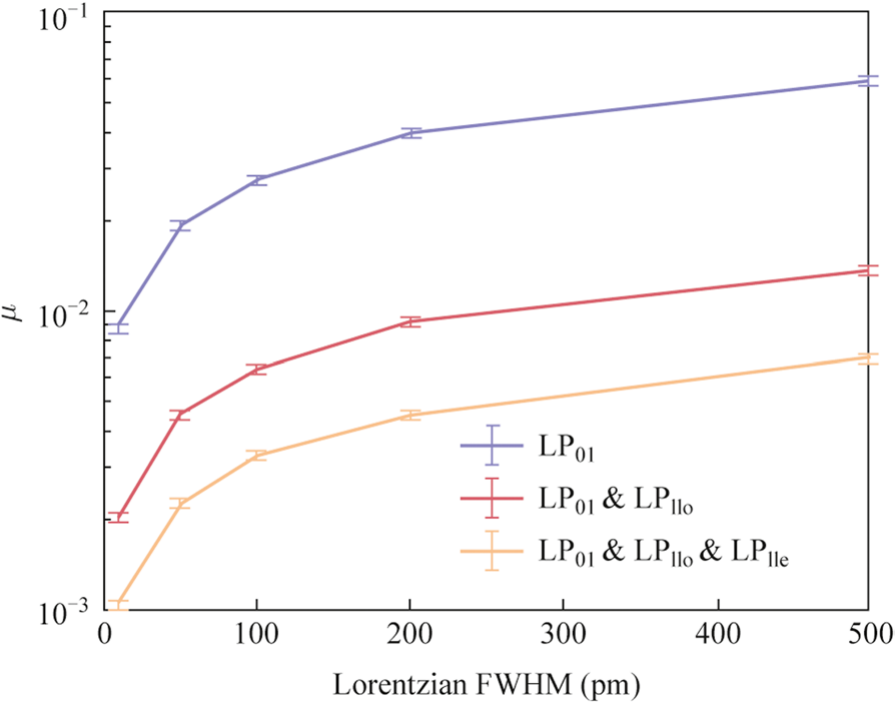
Reconstruction error of spectra with varying Lorentzian FWHM linewidth by using single-mode-excitation, two-mode-excitation, and three-mode-excitation. Error bars in the graph refer to the standard deviation of *μ* in different reconstruction cases

Figure 4 depicts the spectral reconstruction errors obtained from various schemes when applied to spectra with differing Lorentzian FWHM linewidth values. All schemes exhibited a proportional increase in reconstruction error as the FWHM linewidth value increased, which is consistent with previous findings in Ref. [16]. The three-mode-excitation scheme demonstrated the lowest reconstruction error across all scenarios. Specifically, when compared to the single-mode and two-mode excitation methods, the three-mode-excitation method resulted in an average reduction in reconstruction error of 88% and 50%, respectively.

The bandwidth we present currently is relatively narrow, and it can be further increased to dozens of nanometers, considering the tunable laser for calibration can cover such a large bandwidth. However, detecting spectra with wide bandwidth and high resolution simultaneously remains a huge challenge. For speckle RSs, bandwidth and accuracy are always closely related. When the measurement bandwidth is increased, the reliability of the recovery spectra will decrease, hindering their wider acceptance among users [17]. This is because we assume broad-band speckle results from the incoherent superposition of speckle intensity across various channels. The overall speckle contrast decreases inversely with the square root of the number of independent speckle intensities. Achieving accurate spectral reconstruction necessitates a higher speckle contrast than the measurement noise level [20]. A wide, reliable bandwidth needs resorting to methods including frequency-domain segmentation [20], high-dimensional speckle field information detection [17], or increasing the number of informative spatial channels through multiple spectral-to-spatial encoding (e.g., increasing the incident mode conditions).

Actually, when the number of incident modes is increased to a certain extent, the reconstruction accuracy will tend to saturate. According to **I = T·S**, **I** will be an *N* × 1 vector, **T** will have dimensions *N* × *M*, and **S** will be an *M* × 1 vector, where *N* is the spatial channel number and *M* is the spectral channel number considered. If instead *K* distinct launch conditions are considered (*K* = 3 in this paper), **I** will be *KN* × 1 and **T** will be *KN* × *M*. In the context of measuring light with a broadband optical spectrum [31], the number of variables (*M*) that need to be resolved may exceed the number of known observational values (*N*). Under such circumstances, obtaining an accurate solution to the equation becomes challenging. However, by augmenting the incident mode conditions, the number of known observations (*KN*) can surpass the number of variables (*M*), leading to a system with an excess of equations. This surplus mitigates uncertainty in ascertaining the intensity for each spectral channel. Consequently, as the value of *K* increases, if the ratio *M*/*KN* is greater than 1 and exhibits a decreasing trend, the spectral reconstruction accuracy will markedly improve. Conversely, when this ratio falls below 1, the rate of enhancement in reconstruction accuracy will progressively decelerate. Therefore, a larger *K* is not necessarily better, considering that switching incident modes also increases the time cost. An appropriate value of *K* signifies a balance among a faithful spectral recovery bandwidth, considerable accuracy, and acceptable switching times.

**Fig. 5:**
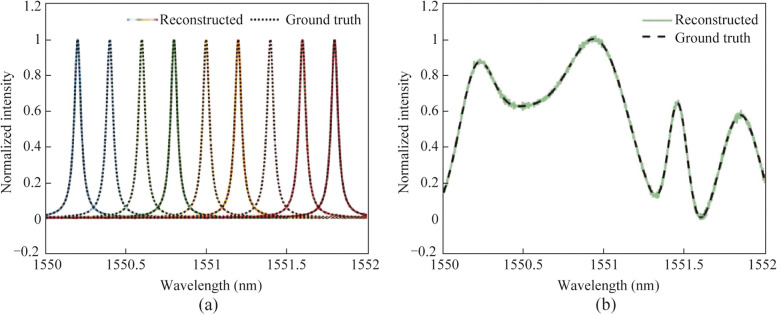
**a** Reconstructed spectra of a set of narrow spectral lines (FWHM linewidth = 0.05 nm) across the entire working range by using the three-mode-excitation method. **b** Reconstructed spectrum for a continuous random signal by using the three-mode-excitation method

The reconstruction of various spectra was performed using the method based on three-mode-excitation. Figure 5a demonstrates a sequence of narrow spectral lines (FWHM linewidth = 0.05 nm) ranging from 1550 to 1552 nm. The average spectral reconstruction error was 0.0022, with the SNR exceeding 20 dB across the entire working range. Figure 5b showcases the reconstructed result for a continuous random spectrum comprising superposed Gaussian kernel functions. The measured spectral reconstruction error was 0.0097.

**Fig. 6:**
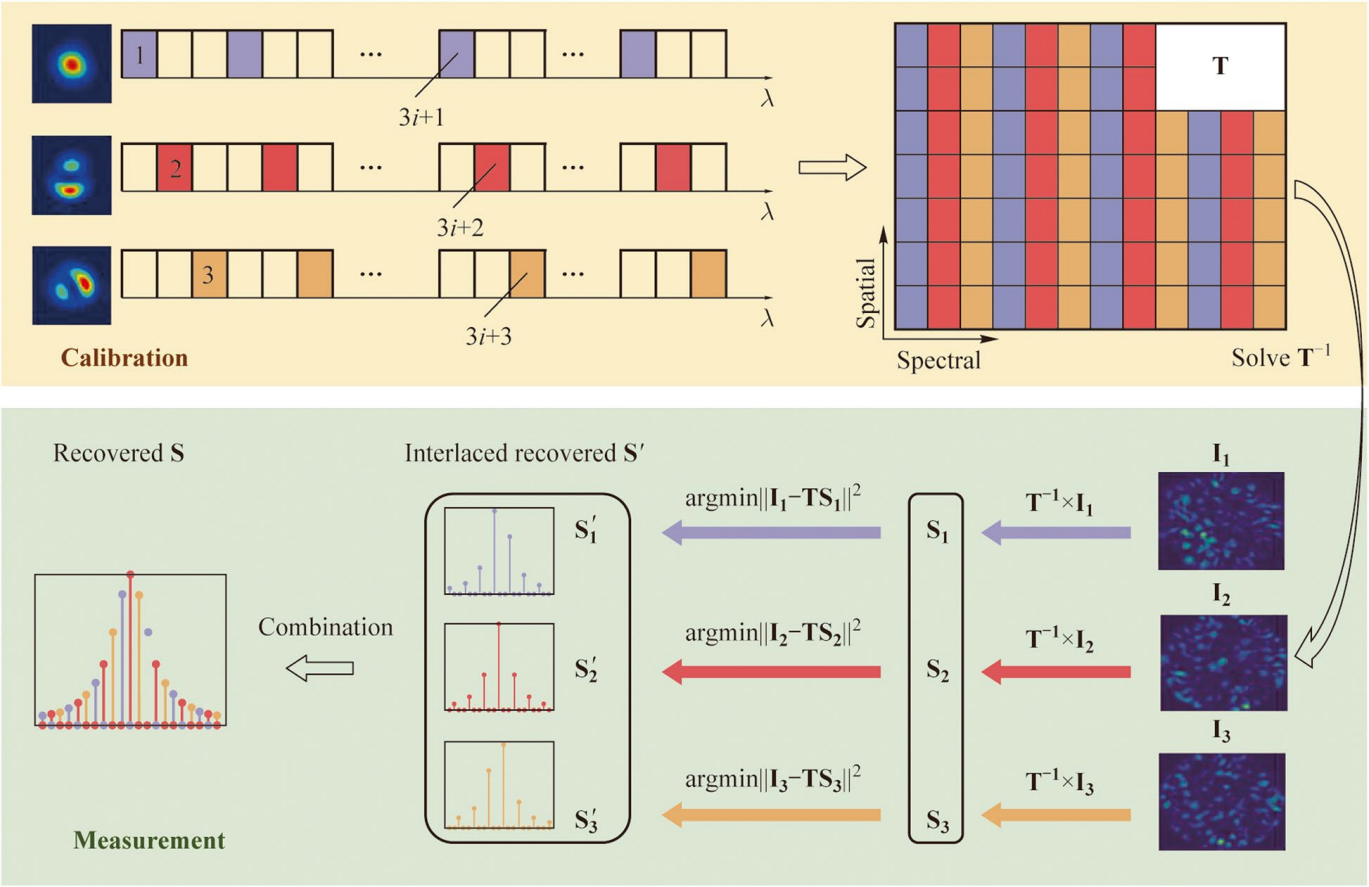
Spectral reconstruction scheme and resolution enhancement principle of alternating mode modulation

Figure 6 illustrates the spectral reconstruction scheme and resolution enhancement principle of alternating mode modulation. During the calibration, different spectral channels were pre-assigned to different launching modes. For example, one custom lookup table can be: the (3*i* + 1)th spectral channel was excited by the LP_01_ mode to obtain calibration speckles (marked in purple in Fig. 6), where *i* = 0, 1, 2,… The (3*i* + 2)th channel was excited by the LP_11o_ mode (marked in red), and the (3*i* + 3)th by the LP_11e_ mode (marked in yellow). During the measurement phase, the test light was injected into the MMF under these three mode conditions, and three corresponding output speckles, **I**_1_, **I**_2_, and **I**_3_ were captured. Three preliminary reconstructed spectra, **S**_1_, **S**_2_, and **S**_3_, were obtained in parallel by multiplying the inverse of **T** with corresponding speckle patterns. Subsequently, an optimization algorithm was employed to minimize ‖**I**_*l*_–**TS**_*l*_‖_2_ (*l* = 1, 2, 3), achieving the optimized spectra **S**_1_^′^, **S**_2_^′^, and **S**_3_^′^. ‖·‖_2_ refers to the calculation of 2-norm. At this point, **S**_1_^′^, **S**_2_^′^, and **S**_3_^′^ were interleaved, with intensity values in some spectral channels approaching zero. Actually, only spectral channels with excitation modes matching those of the measured speckles could reconstruct nonzero values. Taking the reconstruction of **S**_1_^′^ as an example, **I**_1_ was obtained through excitation by the LP_01_ mode, so only the (3*i* + 1)th spectral channels of **S**_1_^′^ could yield nonzero solutions. **S**_1_^′^, **S**_2_^′^, and **S**_3_^′^ were then concatenated to obtain the final reconstructed spectrum, **S**.

**Fig. 7:**
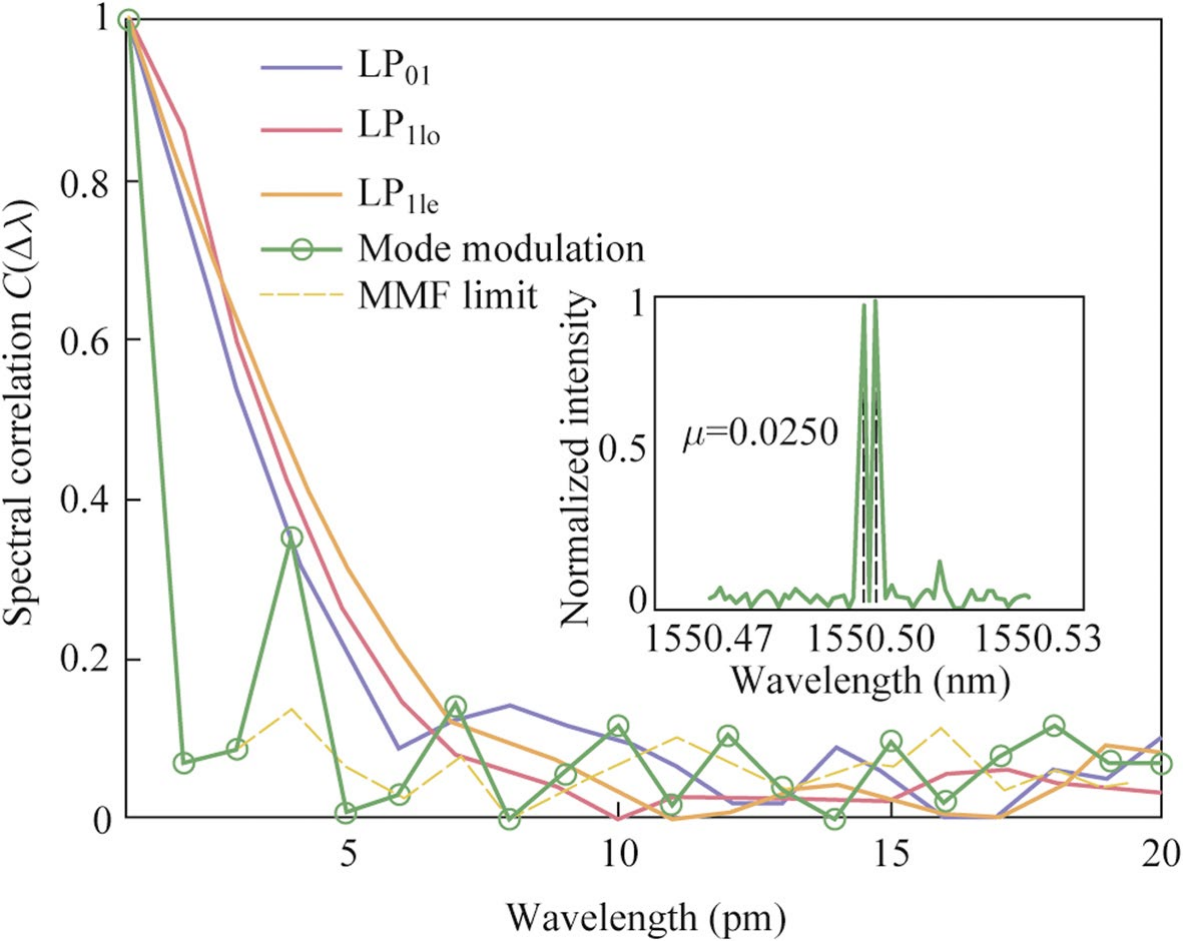
Spectral correlation curves of the cases without mode modulation (all spectral channels excited by LP_01_ mode, LP_11o_ mode, or LP_11e_ mode), the case with mode modulation, and the MMF limit case. The inset illustrates the reconstruction outcomes of two spectral peaks separated by 2 pm using the mode modulation approach. The reconstruction measured 0.0250

As the output speckle field of the MMF depends on both the input wavelength and mode field, this method enhances the differences between adjacent columns of the calibrated transfer matrix **T** through active interleaved coding. Figure 7 shows the spectral correlation curves of the cases without mode modulation (all spectral channels excited by the same mode, i.e., LP_01_, LP_11o_, or LP_11e_ mode), the case with mode modulation, and the MMF limit case.

The spectral correlation function of the speckles can be expressed as:

3 $$C(\Delta \lambda ,x) = \left\langle {\frac{{\left\langle {I(\lambda ,x)I(\lambda + \Delta \lambda ,x)} \right\rangle_{\lambda } }}{{\left\langle {I(\lambda ,x)} \right\rangle_{\lambda } \left\langle {I(\lambda + \Delta \lambda ,x)} \right\rangle_{\lambda } }} - 1} \right\rangle_{x} ,$$

where *I*(*λ*,*x*) is the intensity measured at a position *x* for wavelength *λ*. < ··· > represents the average across wavelength or position ranges. The correlation function value was then scaled to 1 at Δ*λ* = 0. The spectral resolution could be approximately estimated by determining the half-width at half-maximum (HWHM) of the correlation function. We simulated the resolution limit of MMF RSs by exciting all guided modes of an MMF with a core size used in experiments. This resulted in a spectral correlation curve with an HWHM of 1 pm, limited by the wavelength step size in our simulation. Experimental results showed that the HWHM in the absence of mode modulation is approximately 3 − 4 pm. When employing mode modulation, the spectral correlation curve displayed distinct periodicity. The HWHM could be compressed to 1 pm, achieving the theoretical resolution limit of the MMF. This limit was dictated by the smallest tuning step of the calibration light source utilized in our experiments. In fact, when the incident mode is fixed, the change of speckle with wavelength is gradually slow. However, the variation in speckle due to different incident modes can be sharply abrupt. This occurs because the input mode is shaped by the encoder, leading to the magnification of pattern discrepancies. The inset of Fig. 7 demonstrates the reconstruction results of two spectral peaks separated by 2 pm using the mode modulation scheme, with a reconstruction of 0.0250.

## Conclusion

To summarize, we demonstrated a high-accuracy RS based on MDM technology. The MDM involved a custom-made 3 × 1 mode-selective, all-fiber photonics lantern to launch distinct spatial modes into the MMF. This enabled the transmission enhancement of information by expanding the light scattering process, thereby encoding the spectrum more comprehensively into speckle patterns. The proposed RS showed that an impressive spectral resolution of 2 pm and 2000 spectral channels could be recovered. The experimental results indicated that by using more speckles induced by different exciting modes, added independent spatial channels were obtained and thus the spectral reconstruction accuracy could be increased. Exactly, compared to methods using single-mode-excitation and two-mode-excitation, the three-mode-excitation method reduced the recovered error by 88% and 50%. We also proposed a method to enhance resolution by modulating modes alternately, reaching the MMF limit for the HWHM of the spectral correlation function. Future work will focus on further increasing the number of exciting modes to enhance the reconstruction accuracy, as well as studying the trade-off relationship between the widened authentic bandwidth, reconstruction accuracy, and time consumption. In conclusion, this RS was not only concise and highly efficient but also suitable for various high-accuracy, high-resolution spectral measurement scenarios.

## Data Availability

The data that support the findings of this study are available from the corresponding author, upon reasonable request.
